# A COVID-19 University-Based Dental Clinic Experience and Infection Control Protocol Modification for Safe Clinical Education

**DOI:** 10.1055/s-0042-1757467

**Published:** 2022-11-09

**Authors:** Hanadi S. Lingawi, Salwa A. Aldahlawi, Ibtesam K. Afifi

**Affiliations:** 1 Department of Preventive Dentistry, College of Dentistry, Umm Al-Qura University, Makkah, Saudi Arabia; 2 Department of Basic and Clinical Oral Sciences, College of Dentistry, Umm Al- Qura University, Makkah, Saudi Arabia; 3 Department of Medical Microbiology and Immunology, Faculty of Medicine, Tanta University, Egypt

**Keywords:** COVID-19, dental education, clinical training, contact tracing, infection control

## Abstract

**Objectives** The aim of the study was to share our experience of the development and application of a modified infection control protocol at the Dental Teaching Hospital, Umm Al-Qura University (UQUDENT) during the second wave of the COVID-19 pandemic. A second aim was to evaluate the impact of the implemented strategies on preparations for future requirements in clinical dental education.

**Materials and Methods** In this descriptive study, we evaluated the challenges facing dental practice and categorized them into four domains: challenges facing dental practice during the pandemic, the risk of acquiring COVID-19 infection, the design of student clinics, and the financial challenges. The impact of strategies established to deal with such challenges was studied by comparing the number of treated patients from September 1, 2020, to March 01, 2021, with the number treated during the same months pre-COVID-19. The COVID-19 polymerase chain reaction (PCR) confirmed students and health care workers (HCWs) were expressed in numbers and percentages in each category of the study group.

**Results** Policies were set up to deal with the challenges in each domain, after training all the hospital personnel in mitigation of the spread of infection within the hospital. We left a non-working clinic between every two operating clinics, and the patient risk was assessed by triage scoring and health status checks by a Saudi mobile application at the entrance. The hospital delivered more personal protective equipment and obligated all students and HCWs to wear KN95 or N95 masks during procedures. Over 1,500 patients were treated during the study period with more than a 30% reduction in comparison to those in the pre-COVID-19 period, but only 20 UQUDENT personnel had confirmed COVID-19 infection, and all proved to be community-acquired by contact tracing.

**Conclusion** The measures implemented in this study proved effective. With the challenges and limited resources, UQUDENT managed to resume the operation of its dental clinics and training while preventing cross-infection, and it ensured that dental students graduated with the required competency. Sharing experiences between educational institutes will help to graduate safe competent practitioners.

## Introduction

As the World Health Organization (WHO) declared the pandemic of the novel SARS-CoV2 infection in early 2020, COVID-19 became a major public health challenge worldwide, and governments were urged to take serious action. 1 Physical distancing, wearing face masks, and hand hygiene were listed as the top infection control measures to prevent further virus spread and help control the pandemic. However, COVID-19 continued its global sweep, forcing many countries to lock down as a precautionary measure for contact suppression. 2 3 4 Saudi Arabia was among the first countries to implement early precautionary measures aimed at preventing COVID-19 introduction into the country or lessening its impacts if introduced. Suspension of entry to all international Umrah, pilgrims, and tourists, as well as a ban on inbound travel of individuals from COVID-19-affected countries, were implemented before March 2, 2020, when the first COVID-19 case in the country was reported. 5

Worldwide, the impact of the pandemic affected all aspects of life, including education. For general safety, most educational institutes were locked down, 6 meaning that lectures, clinical teaching, and assessments could no longer be conducted as usual, and educational activities shifted to video conference platforms, such as BlackBoard, WebEx, and Microsoft Teams. 6 7 8 On March 18, 2020, in response to the first COVID-19 wave, the Saudi Ministry of Education (MOE) announced closure of all schools and universities, with an immediate shift to an e-learning/distance learning system. 5 9 This included dental training as well.

The nature and characteristics of dental settings involve exposure to the patient's saliva, blood, body fluids, and aerosols; therefore, dental students, faculty members, other healthcare workers (HCWs), and patients were placed at an increased risk of cross-infection and the spread of COVID-19. 8 For this reason, dentistry was listed among the professions with the highest risks of COVID-19 infection and transmission. 10 This high risk prompted the closure of most dental institutes/clinics, with dental services worldwide limited to emergency dental treatment only. 11 12 13 One study investigating the immediate response of the European Dental Institutions showed that the vast majority of dental schools reported using online tools for teaching, with no clinical activities at all; only a very few institutes allowed limited clinical activities. 11

Dental clinical teaching differs from that of other healthcare professions, as students in other training programs observe licensed clinicians to learn how to provide care for patients. By contrast, dental students, very early in their undergraduate years, perform irreversible treatment procedures themselves on patients under the supervision of licensed clinical teachers, who take responsibility for the student's work. Therefore, clinical chair-side teaching sessions with patients are fundamental in the dental curriculum. Finding suitable alternatives in light of the challenges related to the COVID-19 pandemic was therefore critical to ensure that dental students received the proper training to become safe practitioners upon graduation. Many dental associations and health governing bodies offered general guidelines for use in dental practices during the pandemic, but no official recommendations were made for dental educational institutions. 6 14 This forced dental educational institutes to make their own decisions based on the regulations and recommendations provided by their local authorities. Consequently, the specific changes made to the implementation of dental education and the impact of these changes on the outcomes of dental educational programs have varied widely. 15 16

During the pandemic, different dental institutes around the world made different decisions and took diverse approaches toward clinical education. For example, Australia, Hong Kong, Japan, Malaysia, Philippines, Thailand, Pakistan, the United Arab Emirates, Switzerland, and the USA suspended all educational clinical activities, apart from dental emergency treatments, at dental school clinics. 8 17 18 In France, dental students lost about 4 months of clinical practical training due to the cancellation of student clinics and clinical examinations, 19 while training of dental students in Italy was delayed until the following semester due to dental clinic closures at the peak of the pandemic. 20 In China, dental schools postponed clinical examinations, and dental students had to attend additional months of clinical training before graduating. 21 In Saudi Arabia, dental practices were allowed to reopen in July 2020 (during the second COVID-19 wave) by following strict guidelines from the Ministry of Health (MOH). However, clinical dental teaching sessions did not restart until September 2020.

The aim of the present study was to share our experience of applying a modified infection control protocol developed at the Dental Teaching Hospital of Umm Al-Qura University in Makkah, Saudi Arabia, to ensure the safe reopening and operation of the clinical teaching sessions during the second wave of the COVID-19 pandemic. A further goal was to evaluate the impact of the implemented strategies in preparation for the future requirements of clinical dental education.

## Materials and Methods

This descriptive study explored the strategies implemented at the Dental Teaching Hospital, Umm Al-Qura University (UQUDENT), Saudi Arabia, to overcome the challenges faced during the second wave of the COVID-19 pandemic. These strategies were implemented after the reopening of dental clinics but before vaccine availability (i.e., from September 01, 2020 to March 01, 2021). As this study did not include any patient's data, ethical approval was not required.

The strategies were developed after reviewing global guidelines for reopening dental clinics. 15 16 22 Challenges for implementation at UQUDENT were identified and categorized into four main domains: (1)challenges facing dental practice during the pandemic, (2) risk of acquiring COVID-19 infection, (3) the design of student clinics, and (4) financial challenges.

### The First Domain: Challenges in Dental Practice

These challenges involved the adoption of the standard operating procedures and policies recently developed by the MOH for dental practice in Saudi Arabia 6 months after locking down and suspending dental practice. Additional challenges in dental practice included keeping up with the rapidly changing information about the mode of transmission and symptoms of the disease and the lack of previous experience in managing a pandemic.

### The Second Domain: Challenges Posed by the Risk of Acquiring COVID-19 Infection

These challenges explored ways to deal with the nature of dental procedures, which require close proximity between dentists and patients. The risk escalates during aerosol-generating procedures (AGPs), such as fillings, endodontic treatments, and ultrasonic scaling. Possible contact with asymptomatic COVID-19 or colleagues patients was a further challenge that increased the risk of COVID-19 infection.

### The Third Domain: Challenges Regarding the Students' Clinic Design

These challenges considered the limited clinical space, the open floor plan design, absence of air filtration facilities, and confined clinic working hours (8 hours daily, from 8:00 a.m.–4:00 p.m.).

### The Fourth Domain: Financial Challenges

These challenges included handling the limited resources for personal protective equipment (PPE), especially N95 masks, and the increased demands for disinfectants and hand sanitizers.

The impact of the implemented strategies was studied by comparing the number of treated patients during this period with the number treated during the same months pre-COVID-19 from September 01, 2019 to March 01, 2020, considering the numbers of positive COVID-19 students and HCWs.

## Results

The strategies established, to face the challenges detected, were developed after reviewing global guidelines for reopening dental clinics. 16 17 23 The challenges in the first domain mainly involved dealing with the nature of dental practice during the pandemic in preparation for reopening of the dental clinic in September 2020. The infection control committee established policies in accordance with the latest international guidelines and the Saudi MOH protocols. “The policies detailed the dental practice infection control measures taken during the COVID-19 pandemic. The lack of previous experience in managing a pandemic was overcome by conducting orientation and training lectures to explain the newly developed policies for treating dental patients. The trainees included undergraduate and graduate students, dental assistants, and faculty members. Completion of the online lectures was a mandatory requirement before resuming dental clinics. The lectures were also recorded and accessible for students and staff at any time. Training on the proper PPE donning and doffing procedures was conducted for all staff and students with special emphasis on the appropriate doffing of N95 masks to allow for the extended use protocol. Following the MOH recommendations, cleaners were trained in the methods of environmental cleaning and disinfection during the COVID-19 pandemic. A high level of awareness was maintained by having the hospital information screens play WHO and MOH awareness videos. Instruction signs were also distributed in all dental hospital amenities to encourage social distancing, hand hygiene, and face masks.

The challenges in the second domain were addressed by advising all faculty members, students, and employees to download the Saudi COVID-19 mobile applications, Tawakkalna and Tabaud, to create a healthy environment within the dental teaching hospital. The Tawakkalna application was developed by the Saudi government and provided many services during COVID- 19. Among the services provided was individual health status and COVID-19 risk. COVID-19 status was color-coded and categorized as confirmed case, suspected case, or non-infected. The codes were one of the four; the green color means no record of infection (no risk); the orange color represents contacts who must adhere to home isolation/quarantine (moderate risk). The yellow color represents contacts permitted to return to work while taking preventive measures (low risk), and brown for the infected (highest risk). The Tabaud application notifies users if they have come in contact with a COVID-19-positive patient.

**Fig. 1:**
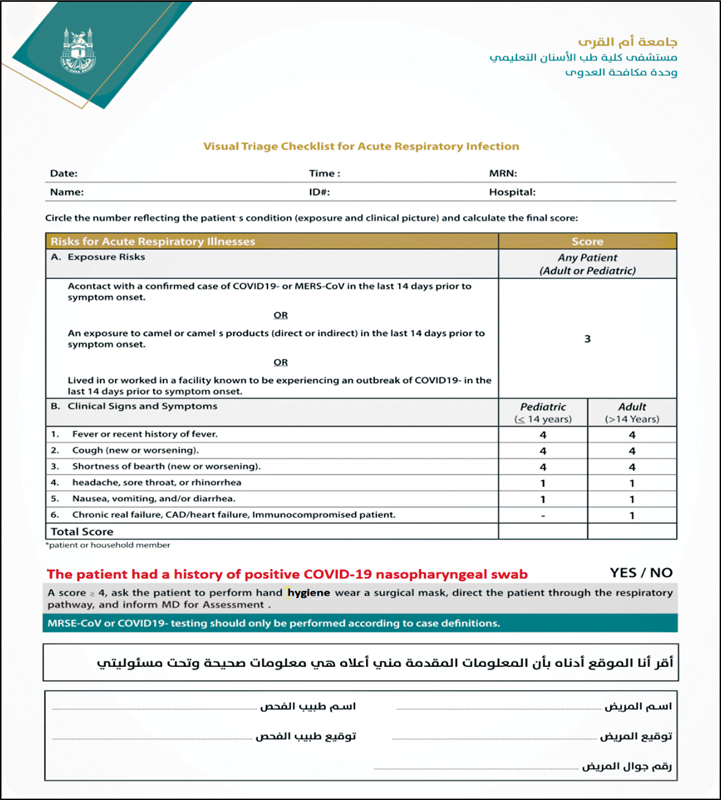
Modified triage form for the Saudi CDC: Coronavirus Disease COVID-19 guidelines, v1.3. (modifications are highlighted in red print).

A triage station (visual screening) was also established at the dental hospital entrance. The infection risk was assessed by measuring body temperature and completing a modified MOH triage form. Questions about a history of positive COVID-19 infection or nasopharyngeal swab test were added to the form. The person's signature, national identification number, and contact information, including phone number, were also obtained ( Fig. 1 ).

Entry and exit pathways were demarcated for patients and students to preserve social distancing and prevent crowding. Seats in the waiting areas were marked to guide places to sit for social distancing. Extra hand sanitizer dispensers were made available in the hospital reception and clinics.

**Fig. 2:**
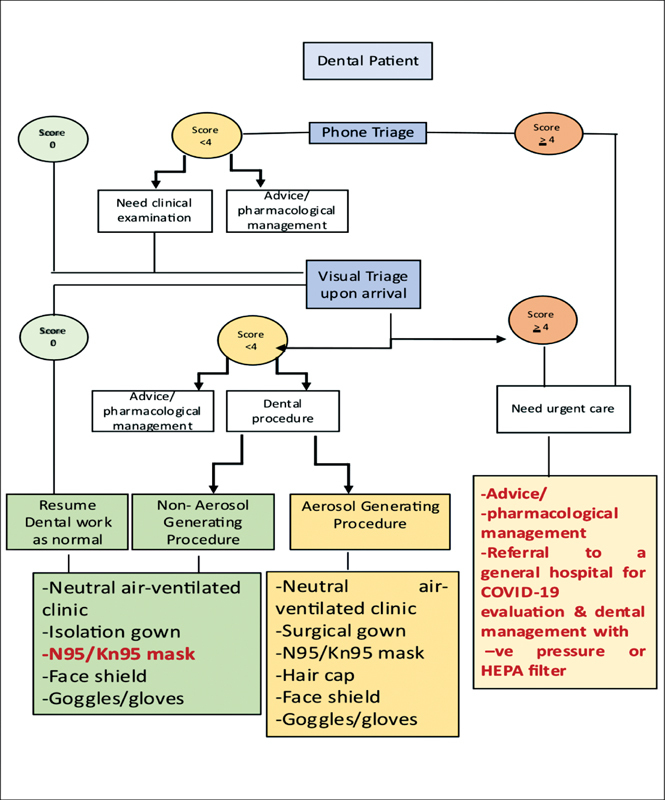
Protocol for providing dental treatment at UQUDENT during the second wave of COVID-19. The MOH guidelines for reopening dental services in governmental and private sectors during the COVID-19 pandemic were modified, taking into consideration the UQUDENT clinical area design and available resources (modifications are highlighted in red print).

The implemented protocol for patient treatment was in accordance with the MOH guidelines. However, those guidelines were modified to consider the clinic design and the available resources ( Fig. 2 ). Students were instructed to wear PPE, including high-efficiency N95 masks and face shields, while treating patients, regardless of the dental treatment provided.

**Fig. 3:**
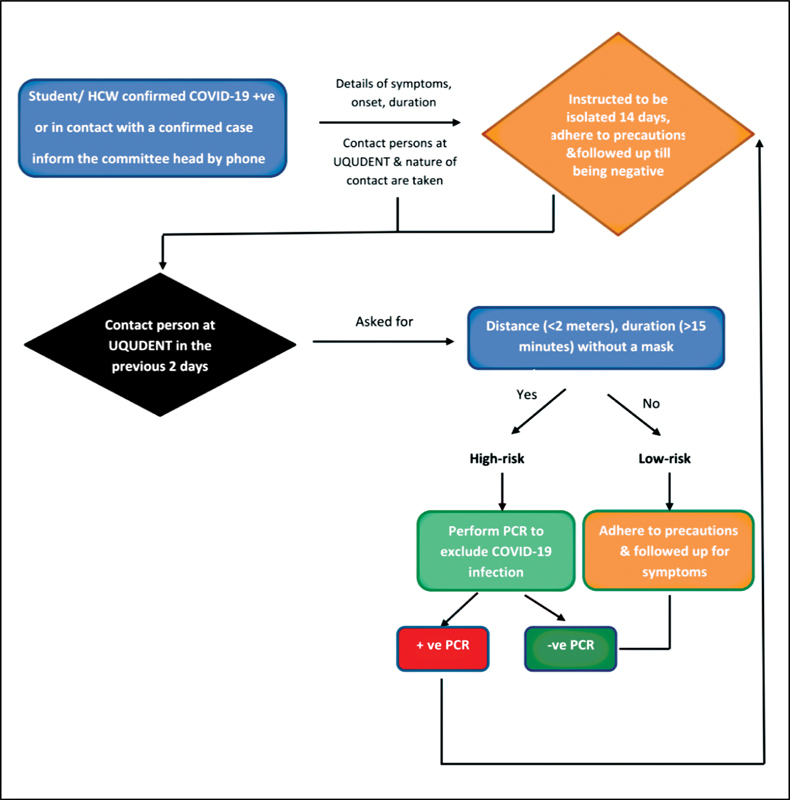
Contact tracing protocol implemented by the committee at UQUDENT.

A COVID-19 contact tracing committee was established under the supervision of the infection control unit. Its aim was to receive reports from students, dental assistants, employees, or faculty members who had acquired COVID-19 infection or came in contact with confirmed or suspected COVID-19 patients. The committee tracked all individuals in contact with the case during their presence at UQUDENT and then determined the risk and guidelines for limiting the spread of infection, based on the protocol presented in Fig. 3 . Contact tracing remained in effect until the person showed a negative PCR test or a lack of symptoms. Table 1 presents the number of UQUDENT students, faculty members, dental assistants, and auxiliary staff who were confirmed positive for COVID-19 by PCR from September 01, 2020 to March 01, 2021.

**Table 1:** The numbers and percentages of UQUDENT confirmed PCR-positive tests for COVID-19 from 1/9/2020 to 1/3/2021

	Students						Faculty Members (112)	Dental Assistants (20)	Auxiliary Staff (14)
	Undergraduate ( *N* )			Postgraduate ( *N* )					
Level (total number)	Year 4 (47)	Year 5 (54)	Year 6 (55)	Interns (64)	Graduate Residents (7)	Dental Assistant Program (23)			
Number of COVID-19 cases (%)	5 (10.6%)	4 (7.4%)	1 (1.8%)	1 (1.56%)	0 (0%)	1 (4.3%)	2 (1.78%)	0 (0%)	4 (28.57%)
**Total 18/396 (4.54%)**									

**Fig. 4:**
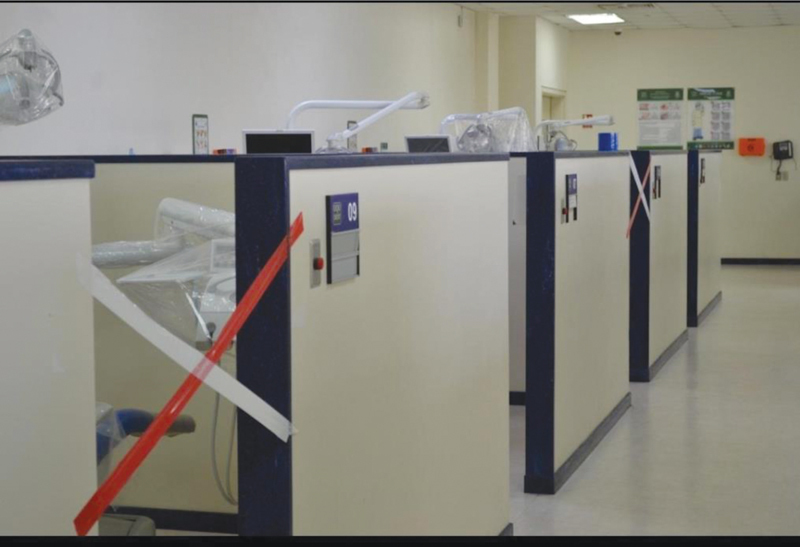
Non-working clinic between every two operating clinics.

The challenges in the third domain regarding the student clinic design and open floor plan were dealt with by social distancing between students, for their safety, and the safety of patients during treatment by leaving non-working clinics between every two working clinics ( Fig. 4 ). This decreased the number of working clinics, so the students in each level were divided into two groups to work alternately. The clinic schedule was extended from 8 to 12 hours (8:00 a.m. to 8:00 p.m.) to accommodate all the student groups. A fallow time of 1 hour was allowed between sessions to allow aerosols to settle before cleaning and disinfecting the unit.

The challenge of the absence of air filtration facilities was resolved by having patients with suspected COVID-19 and a triage score ≥ 4 referred to the COVID-19 treatment facility.

Strict implementation of the stated infection control policies was ensured by scheduling members of the infection control team for auditing and follow-up using a specially designed checklist. Any violations were reported to the head of the infection control unit for appropriate action.

The challenges in the fourth domain, financial challenges, were rectified by the provision of urgent materials required for infection control implementation during the COVID-19 pandemic by the administration. These materials included N95 and KN95 masks, face shields, hand sanitizers, and hanging sanitizer dispensers. The Saudi MOH policy of extended N-95 mask use was also implemented. That policy recommended five uses of the same respirator by the same HCW, provided that the mask was covered by a changeable surgical mask or face shield when performing AGPs.

## The Impact of the Implemented Strategies

The number of treated patients pre-COVID-19 (from 1/9/2019 to 1/3/2020) and during the second wave (1/9/2020 to 1/3/2021) after implementing policies to overcome the challenges were compared.

Table 2 shows that the number of treated patients was reduced during the second wave of COVID-19. Exceptions were cases treated in the specialty and postgraduate clinics, which increased from 130 patients pre-COVID-19 to 268 patients during the second wave.

**Table 2:** Comparison between the numbers of treated patients pre-COVID-19 and during the second wave (1/9/2020 to 1/3/2021)

Number of treated cases at UQUDENT							
Undergraduate student cases		Specialty and postgraduate cases		Emergency cases		Total	
Pre-COVID-19	During the second wave	Pre-COVID-19	During the second wave	Pre-COVID-19	During the second wave	Pre-COVID-19	During the second wave
1,619	1,119	130	268	735	158	2,484	1,545

## Discussion

We described in detail the experience of UQUDENT in the development and application of a modified infection control protocol to ensure the safe reopening and operation of our dental clinics during the second wave of the COVID-19 outbreak. Our experience may provide guidance to other dental schools with similar environments and facilities in planning their clinical educational operation during this pandemic or future outbreaks.

The main aim of any dental school is to assure quality clinical training of dental students while maintaining the safety of the students, staff, and patients. Ensuring that clinical-year students received the required training and overcame the period they lost during the complete lockdown at the early phase of the COVID-19 pandemic was challenging. In addition, many patients had their dental treatments interrupted and delayed during the lockdown period, and many had setbacks in their dental status. When the dental clinic reopened, most of these cases needed reassessment, and some treatments had to be re-done. 12 23

Reopening the dental clinics at UQUDENT required the expansion of infection control policies and procedures based on local authority guidelines. 16 However, the guidelines also required modifications to allow proper implementation that considered the financial and environmental constraints. For example, the UQUDENT clinics have an open floor plan in which short walls separate dental operatories to maintain patient privacy. Dental procedures produce splatter and aerosols contaminated by blood or saliva. 24 Both splatter and aerosols can transmit pathogens some distance away from the main treatment area. However, the literature is unclear regarding how far aerosols can spread. 24 25 26 27 The SARS-CoV-2 virus has been isolated from the saliva of infected patients, 28 including asymptomatic individuals, 29 but the potential viral load in the aerosols produced by dental procedures is unknown. Initial studies have also indicated that the virus could remain viable for hours in the aerosols and for days on surfaces. 30 However, no effective engineering control has been proven to eradicate aerosols at the point of generation or to eliminate the risk of aerosol transmission of viral diseases. 31

Negative pressure rooms have been recommended to reduce the risk of infection by aerosols, 22 but these rooms are not widely used in dental clinics. Alternatively, air filtration systems that use high efficiency particle air (HEPA) filtration were suggested by the US Centers for Disease Control (CDC), 22 as these units can remove up to 99% of airborne pathogens in just 23 minutes, thereby decreasing room turnover time. 32 Dental schools opted either to conduct AGPs in closed operatories with negative pressure 33 or increase the room turnover time. 34 A dental school in Malaysia followed another protocol whereby AGP treatments were provided in enclosed pods that used specially designed physical barriers 2 m in height around each cubicle. 35

At UQUDENT, minimizing AGPs was encouraged by considering the use of hand instrumentation and minimally invasive dentistry techniques whenever possible. The use of rubber dams, high volume suction, and other precautions to decrease aerosol generation was also emphasized. 36 The challenge posed for containing the spread of aerosols in the open-plan clinic was addressed by providing one non-working clinic between each two operating clinics, thereby increasing the distance between operating clinics to 3 m to help minimize the cross-infection risk. 37 Another consideration was to increase the time interval between patients to 1 hour to allow aerosols to settle before physical cleaning and disinfection of the clinic. Although the recommended time applied for aerosol settling was variable, 38 recent studies indicate that aerosol settling mostly occurs in the first 10 minutes after the end of a procedure. 37 The protocol implemented at UQUDENT reduced the number of patients treated at each session and increased the treatment time needed. In fact, the number of patients treated at the undergraduate clinic was 30% lower than the number in the same period in the previous year. However, the number of patients treated by specialists and graduate residents almost doubled, reflecting increased referrals to specialized care.

The patient risk of being exposed to COVID-19 was assessed by requiring patients to fill out a triage form before entering UQUDENT. The triage form had many limitations, including its dependence on self-reporting and honesty of the patient and its failure to identify asymptomatic or pre-symptomatic subjects. 29 However, it helped to categorize the patient's risk. For example, those scoring < 4 were categorized as low risk. Those determined as high risk were either provided with pharmaceutical management or referred to a COVID-19-ready facility. We modified the MOH triage form by adding questions about any history of positive PCR for COVID-19 in the previous 14 days. The patient's national identification number, signature, and contact information were also included to help with contact tracing in the event of exposure.

Some dental schools required patients to provide a negative PCR test before treatment 39 ; however, UQUDENT relied on the Saudi Twakkalna Application. The Twakkalna provided a reliable way to confirm the health status of patients, students, and employees, as it is directly connected to their official health records through the Saudi Ministry of Health 40 . In addition, the individual risk of COVID-19 exposure was color coded and labeled as confirmed COVID-19 case (by PCR), exposed or suspected COVID-19 case (in contact with a confirmed COVID-19 or history of travel, etc.) and not being infected or in contact with a case. The information on Twakkalna app was updated daily, thereby serving as an additional validation of health status. Aga Khan University in Pakistan also used a similar approach involving an interactive chatbot application that was filled out by each employee every day to determine the infection status. 41 Positive impact of digital technologies on patient care was reported in the literature. 42 The increased use of telehealth during the pandemic was only possible due to the enhancement in information and communication technologies that allowed patients to access health services remotely. 43 Various applications were developed not only for online consultation, and medical appointment booking but also for awareness building, sharing information, symptom monitoring, and contact tracing. 40 However, further research on various application effectiveness on mitigating and containing the spread of the virus while addressing privacy and safety concerns is needed. 44

Another challenge arose due to the decreased number of working clinics per session and lowering of the student/supervisor ratio, as this resulted in extending the clinical hours into the evening to accommodate all the required training to clinical year students. This has increased the workload of both academic and auxiliary staff, and in the long term, it may end in a shortage in manpower.

Limited clinical space and the increased costs of specific PPE and infection control added to the increased burdens on the resources in a demanding field of study. We avoided shortages of particulate respirators (N95 and KN95 masks) by following the MOH policy of the extended use of the same respirator for up to five uses. A secondary surgical mask and a face shield were used to cover the N95 and were changed after each patient. 45

The implementation of modified infection control policies achieved many advantages. The students become more efficient in utilizing the clinical time allocated to them. The attention to minimizing AGPs whenever possible meant that more emphasis was placed on preventive treatment and minimally invasive dentistry. The infection control team empowered maximal cross-infection control for the safety of everyone. Many innovative technologies were utilized, such as teledentistry, electronic triage, and contact tracing, and these enhanced the patient experience.

The clinical protocols applied in UQUDENT were confirmed effective, considering the fact that the city of Makkah was one of the Saudi Arabian hot spots of COVID-19 infection, with more than 1,272 cases reported in the city from September 2020 to March 2021. 46 To the best of our knowledge, none of the published articles that presented treatment protocols for operating dental clinics during the COVID-19 pandemic have reported on the effectiveness of those measures. 47

The UQUDENT hospital database recorded over 1,500 patients treated during the period from September 2020 to March 2021. However, the contact tracing committee reported that fewer than 20 (4.5%) UQUDENT personnel had confirmed COVID-19 infections during the same period. All cases were community acquired, and their tracing reports and 14-day follow-ups for their contacts at UQUDENT confirmed that no cross-infection or virus transmission occurred to any of the students, hospital staff, or patients. This validates that the strict infection control protocols and risk-mitigating measures taken by the school effectively prevented infection transmission. As an alternative protocol for contact tracing, Aga Khan University provided a hotline for patients to call if they developed symptoms or tested positive for COVID-19 after receiving dental treatment. 41 However, they did not perform contact tracing for contacts from students and HCWs at their hospital as UQUDENT did.

The Future Perspective

Dental clinic operations may never return to the pre-COVID normal situation. Even with widespread vaccination programs, the risk of a future outbreak of infection is still high, as SARS-CoV-2 had mutated into more than 80 variants since the outbreak, and the long-term immunity provided by vaccines against the virus or its variants remains in question. 48 Infection control has always been an integral part of dental practice, and dental guidelines have been frequently updated in response to emerging diseases from the beginning of the 19th century until now for practice in everyday dental procedures. 49 Therefore, dental schools should be prepared for continuous monitoring and operational procedure changes depending on local COVID-19 case rates to provide safe patient care and safe education.

Farshidfar et al 50 proposed a multilevel system for dental clinic service operation based on local COVID-19 rates, vaccination rates, and testing accessibility. However, collaboration between international dental schools is essential to focus dental education and training on health promotion and emphasize preventive and minimally invasive dentistry.

## Conclusion

Quality training of dental students is the goal of any academic dental institute; however, the safety of the students, patients, and academic and non-academic staff is very important. The measures implemented at UQUDENT proved effective. Despite the challenges and limited resources, UQUDENT managed to resume the operation of its dental clinics and training of its students, while also preventing cross-infection. This has ensured that the dental students will graduate with the required competency to perform as safe independent practitioners.
